# Explicit Stress Communication Facilitates Perceived Responsiveness in Dyadic Coping

**DOI:** 10.3389/fpsyg.2019.00401

**Published:** 2019-02-27

**Authors:** Ariela Francesca Pagani, Silvia Donato, Miriam Parise, Anna Bertoni, Raffaella Iafrate, Dominik Schoebi

**Affiliations:** ^1^Family Studies and Research University Centre, Università Cattolica del Sacro Cuore, Milan, Italy; ^2^Department of Psychology, Family Studies and Research University Centre, Università Cattolica del Sacro Cuore, Milan, Italy; ^3^Department of Psychology, University of Fribourg, Fribourg, Switzerland

**Keywords:** dyadic coping, explicit stress communication, perceived responsiveness, couple relationship, daily diary

## Abstract

The present study was aimed at examining the role of explicit stress communication in the context of dyadic coping. The general aim of the present study was to test (a) whether explicit communication of daily stressful events predicted relationship satisfaction and (b) whether the perception of responsiveness in dyadic coping mediated the association between explicit stress communication and partners’ satisfaction. We analyzed daily diary data from 55 married couples and multilevel analyses suggested that, although explicit stress communication was not associated with relationship satisfaction, it predicted both partners’ responsiveness in dyadic coping behaviors. Finally, responsive dyadic coping behaviors mediated the relationship between explicit stress communication and relationship satisfaction. On the whole, our findings showed that perceived responsiveness in dyadic coping with daily stressors was facilitated by explicit stress communication and that this contributed to the effectiveness of dyadic coping behaviors in fostering partners’ relationship satisfaction. We discussed how the current study contributes to the understanding of the dyadic coping process and its contribution to partners’ satisfaction, underscoring the importance of communication skills.

## Introduction

How a couple deals with stress can have a lasting effect on the relationship, even when responding to daily stressors (Randall and Bodenmann, 2009). A couple’s joint response to a stressor (i.e., dyadic coping -see par. 1.1) can protect relationships from the wear and tear of daily stress, enhance partners’ intimacy and further strengthen the couple relationship (Bodenmann, 2005; Milek et al., 2015). Dyadic coping requires that both partners engage in a communication process when responding to the stressor. Key elements of such communication process include one partner disclosing relevant information about the event, and the stress and negative emotions he/she feels, and the other partner responding to this disclosure (e.g., Reis and Gable, 2015). Once a couple has engaged in such a communication sequence, the partner who initiated the sequence perceives, decodes, and evaluates the other’s reactions, being sensitive to the degree to which the partner responded to their concerns and needs (i.e., to the partner’s “responsiveness;” Reis and Shaver, 1988). Perceptions of responsiveness are an integral part of the process of dyadic coping with stress (Bodenmann, 2007). It is not uncommon, however, that partners fail to be responsive to stress expressions and related disclosures. This can be the result of a lack of motivation or skills, but it can also occur because the partner does not adequately perceive or interpret the disclosed content (Reis and Shaver, 1988; Reis and Clark, 2013; Reis and Gable, 2015). Therefore, we propose that explicit and unambiguous stress communication should inoculate couples against such maladaptive coping dynamics, enhance the partner’s responsiveness in the dyadic coping process, and thereby improve adaptation to the stressor and strengthen the relationship. The current research tests these predictions. In the remainder of the introduction, we first discuss the dyadic coping process and the role of responsive reactions during this process. Next, we point out the relevance of explicit stress communication for partners’ responsiveness, and specifically for responsive dyadic coping behaviors, and partners’ satisfaction with their relationship.

### Dyadic Coping

In everyday life, all couples encounter situations in which they have to cope with minor or major stressors, and doing so effectively helps to maintain well-being and relationship satisfaction. When partners communicate about a stressor to jointly respond to it, dyadic coping occurs (Bodenmann, 1995). Dyadic coping is associated with enhanced relationship satisfaction (for a meta-analytic review, see Falconier et al., 2015) and can protect the relationship from the negative effects of stressful events (e.g., Gasbarrini et al., 2015).

Dyadic coping is conceptualized as a dyadic process involving both partners (Bodenmann, 1995, 1997, 2005), and involving an interplay between one partner’s stress signals and the other partner’s coping reactions (Revenson et al., 2005). The key theoretical framework, the Systematic-Transactional Model of dyadic coping (STM; Bodenmann, 1995, 2005), is based on Lazarus and Folkman’s (1984) stress and coping paradigm and conceptualizes dyadic coping as partners’ participation in the stress-related regulation of emotions and behavior that directly or indirectly concern both partners. The process of dyadic coping can be thought of as circular sequence, in which one partner’s communication of stress (with different levels of explicitness and through various channels) is perceived and evaluated by the other partner (cfr. “dyadic stress appraisal;” Bodenmann, 2005; Bodenmann et al., 2016), who in turn reacts with a dyadic coping response (both in terms of supporting the stressed partner or implementing joint coping strategies)^1^. What the other does or does not during the process is then perceived, decoded and evaluated by the partner who initiated the sequence and it is on the basis of these perceptions and evaluations that the stressed partner may feel more or less satisfied with the response and may continue with the cycle by giving his/her own feedback to the other. Partner responsiveness is a key element of these evaluations. Dyadic coping responses, in fact, have been conceptualized as either positive (i.e., providing emotional or instrumental support) or negative (i.e., unsupportive, ambivalent, or superficial forms of support; see, for a presentation of various types of dyadic coping responses: Bodenmann, 1997, 2005) and -besides their theoretical valence- they can be perceived as more or less responsive to the partners’ needs, which makes them more or less beneficial for the individual and the relationship (e.g., Bodenmann, 2007; Iafrate and Donato, 2012; Donato, 2014).

Dyadic coping reactions that are highly responsive convey that one’s partner is present and committed to the relationship, that he or she can be relied on, and that he or she is also a skillful support provider. It allows both partners to feel a reciprocal connection, enhancing a sense of trust, validation and support, and a sense of we-ness (Cutrona, 1996; Bodenmann, 2005). In other words, such a relationship is marked by high intimacy, with partners attending to and responding “supportively to each other’s needs, wishes, concerns, and goals” (Reis and Clark, 2013, p. 400). Responsive interactions favor partners’ belief that both members of the couple will take care of each other and will react supportively (Reis et al., 2004). Perceived responsiveness is important when partners decide to share personal stressors, or when they want to resolve a conflict, or share or negotiate important personal needs and goals (Reis and Clark, 2013; Reis and Gable, 2015). Specifically, perceptions of partner responsiveness were found to maximize the benefits derived from social support behaviors (e.g., Collins and Feeney, 2000; Maisel and Gable, 2009), which represent an important component of the dyadic coping process. Beyond any specific behavioral response a partner can enact in front of the other stress communication, perceptions of responsiveness are likely to affect partners’ relationship satisfaction as resulting from the dyadic coping process.

Although communication is considered an integral part of the dyadic coping model, less attention has been dedicated to the way partners communicate stressful events, and whether the nature of such a communication might affect whether a partner reacts more or less responsively. While much more attention to the role of communication can be found in the support literature, and particularly in the study of support solicitation (e.g., Cunningham and Barbee, 2000), surprisingly less attention was devoted to this aspect in the dyadic coping literature. In the context of the dyadic coping process, communication actually allows the stressful event to become a relational issue: It is through the communication of the event and of the stress derived from it that partners can engage in dyadic coping reactions (Bodenmann, 2005). Moreover, in the dyadic coping process communication may not only indicate which behavioral options better match with the kind of support that is requested by the stressed partner (cfr. the “optimal matching model;” Cutrona and Russell, 1990), but also allows to make such behaviors as responsive as possible to the others’ needs. The same actions of practical support (i.e., delegated dyadic coping, as define within the STM), for example, can be carried out with more or less attention to the other’s need of protect his/her own sense of autonomy and competence.

### The Role of Explicit Stress Communication for Responsive Dyadic Coping Behaviors

Research on communication suggests that couples in which both partners communicate their feelings and concerns openly reported higher levels of relational satisfaction than couples who communicate without elaborating on the events or their feelings (Guerrero et al., 2011). More specifically, communication may lead to relational happiness if it is characterized by a mutual discussion of problems, by partners’ expression of feelings, by partners’ attempts to understand the point of view of the other, and by a negotiation to find a solution to the problem (Katriel and Philipsen, 1981; Caughlin, 2003; Chi et al., 2013). The central role of explicit communication is highlighted in a recent study on communication of positive events and daily well-being, showing that on days partners reported more explicit communication of positive events, they also reported better individual and relational well-being (Pagani et al., 2015). The authors defined explicit communication as referring to partners talking openly and specifically about an event, adding details about it and possibly expressing their own points of view and emotions with regard to it, while implicit communication to partners talking only indirectly or superficially about the event, without elaborating on it and/or without direct expression of their perspectives and emotional reactions to the event. While explicit communication gives the listener sufficient information to accurately understand what happened and the effects of it on the stressed partner, implicit communication can be ambiguous and lead to misunderstandings. Yet, although partners value open and explicit communication of events and emotions, and although they try to implement it, they often communicate in a closed and implicit way (Kirkman et al., 2005; Caughlin et al., 2011; Goldsmith and Domann-Scholz, 2013), raising the risk that communicated events and moods are misinterpreted or misunderstood.

For effective dyadic coping transactions more specifically, it is not only important that the stressed partner interprets the other’s supportive reactions as responsive to his or her needs, but also that the stressed partner is involved in the dyadic exchange and enables the other to enact responsive behaviors. Limited attention has been paid to the role of the communication component of the dyadic coping process, even though communication is a critical part of dyadic coping trainings (CCET, Bodenmann and Shantinath, 2004; TOGETHER, Falconier, 2015). Some insights on the role of communication can be borrowed from the social support literature (for conceptual differences between dyadic coping and partner support, see Donato, 2014; Donato et al., 2015). In this literature, the contribution of the support recipient to the support process has recently gained increased attention (e.g., Lawrence et al., 2008; Verhofstadt et al., 2013). Support seekers play an active role in support transactions, contributing in important ways to how the interaction evolves (Pearlin and McCall, 1990). Moreover, research on support solicitation has shown that positive vs. negative support seeking behaviors differ between distressed and non-distressed spouses (Verhofstadt et al., 2013). In their broad definition of support seeking behaviors, the authors included explicitness of communication as a key component of positive support seeking and found that “when seeking support, distressed couples are -as compared to non-distressed ones- more inclined to make demands for help, to complain and whine and less inclined to ask for help or state their needs in an open and clear way” (Verhofstadt et al., 2013, p. 334).

Specifically, it has been theorized for the case of dyadic coping that a clear and explicit stress communication is required, so that the other could implement effective forms of coping (Bodenmann, 2005). Only recently, however, has the role of the communication mode received empirical attention. It has been found, for example, that, in couples with one depressed partner, the enhancement of mutual support and explicit communication about the personal stress through coping-oriented couple therapy was positively related to partners’ levels of relationship satisfaction and expressed emotions (Bodenmann et al., 2008). Clear and explicit stress communication is arguably more suited to engage the partner into a responsive interaction than implicit communication (Reis, unpublished), as it helps avoiding ambiguity about the intentions and content of the communication. More recently, additional evidence suggested that during videotaped discussions following a stress induction, partners adjusted their dyadic coping behaviors as a function of the form of stress communication used by the stressed partner (Kuhn et al., 2017). In particular, problem-oriented stress communication consistently predicted problem-oriented dyadic coping, while emotion-oriented dyadic coping was more likely to follow other forms of stress communication (emotion-oriented, non-verbal, neutral; Kuhn et al., 2017). What remained unaddressed is whether explicit stress communication had implications for the perceived responsiveness of the other’s dyadic coping reactions. This seems a crucial link, however, as partners’ perceptions of the other’s dyadic coping behaviors are considered key components of the dyadic coping process, mediating the link between actual behaviors and relationship satisfaction (Donato et al., 2015).

### The Current Study

The general aim of the present study was to examine the role of explicit communication of daily stressful events for facilitating partners’ responsive dyadic coping behaviors and, in turn, sustaining their relationship satisfaction. In particular, we examined whether greater communication explicitness of daily stressful events predicted higher relationship satisfaction (Hypothesis 1). Second, we tested whether perceptions of more responsive dyadic coping reactions from the partner mediated the association between the stressed partner’s communication of his or her stress and his or her own relationship satisfaction (Hypothesis 2). We focused on explicitness of disclosures about the stressful event (i.e., event stress communication), rather than explicitness in the expression of the emotions connected to the event, given that explicit emotion communication was found to be rare in dyadic coping interactions (Kuhn et al., 2017). Finally, we tested whether the above associations differed by partners’ gender. We did not have specific hypotheses at this regard, as research on both stress communication and dyadic coping reported relatively inconsistent findings in terms of gender effects. While some research found gender effects in self-disclosure (Dindia and Allen, 1992), more recent research specifically focusing on stress communication did not (Kuhn et al., 2017). Literature on dyadic coping similarly evidenced both gender differences (e.g., Bodenmann et al., 2015) and similarities (Donato et al., 2015) in partner’s dyadic coping responses.

## Materials and Methods

### Participants

Participants were 55 Italian heterosexual couples (*N* = 110 subjects). Age range of partners was 26–64 years (women: *M* = 42.61, *SD* = 7.88; men: *M* = 45.45, *SD* = 8.42). Partners were together for 17 years on average (*SD* = 9.40). About half of all men (49.1%) reported a technical school diploma or a high school diploma, while 43.6% of female partners reported a higher-level degree. The modal net income was between 1,000 and 1,500 Euro for both women (34.6%) and men (38.2%). Ninety-six point four percent of participants were Catholic, while the remaining partners reported no religious affiliation. This feature is in line with the prevalence of Catholic religious affiliation in Italy. Participants were recruited partially through snowball sampling and partially through the help of their children’s school. In particular, an institute (from elementary school to high school) gave us permission to handle questionnaires out to students’ parents, whenever willing to participate to the study. To participate to the study partners had to be cohabiting for at least 3 years.

### Procedure

Couples filled in a time-based electronic daily diary on a *Personal Digital Assistant* (PDA) twice a day (during the lunch break and before going to bed) for 2 weeks. Research assistants visited participants in their home to help them familiarize with the use of the PDAs and the reporting plan. Participants were instructed not to provide reports retrospectively if they had forgotten to complete the questionnaire at the expected time. The device was also programed to prevent returning to previous sets of questions. Participants were informed that participation was voluntary, that they could stop whenever they wanted without justification. Written informed consent was filled in by all couples. The study protocol was not reviewed by the ethics committee, since it was not required at the time of data collection as per University’s guidelines and national regulations. However, it complied with the Ethical Guidelines of the Italian Association of Psychology (AIP) and with the Ethical Guidelines of the American Psychological Association (APA).

### Measures

#### Explicit Stress Communication

Participants were asked to report twice a day (during the lunch break and before going to bed) whether they experienced stressful events and whether they shared them with the partner. In case they shared the event with the partner, we assessed the degree to which the event was communicated explicitly with the item “When I communicated what happened to my partner, to what extent was I clear and explicit?” Participants responded by means of a 5-point scale (from 1 = *not at all* to 5 = *very much*).

#### Perceived Responsiveness

In both daily assessments, in case participants reported they experienced a negative event, they were also asked to indicate their perception of the partner’s responsiveness using the following prompt: “When I communicated this negative event, to what extent my partner….” The prompt was followed by three items based on Reis (unpublished) perceived responsiveness scale: “My partner understood me,” “My partner made me feel like he/she valued my abilities and opinions,” “My partner made me feel cared for.” Participants reported their agreement to each item on a 5-point scale (from 1 = *not at all* to 5 = *very much*). The three items were combined to create average responsiveness scores. Cronbach’s alpha for the scale was 0.93.

#### Relationship Satisfaction

The momentary relationship satisfaction was assessed with an item starting from the stem “Today our relationship was…” and measured on a 7-point scale (1 = *terrible*, 4 = *ok*, 7 = *terrific*). These reports were assessed only once per day before going to bed.

### Data Analysis

Due to the nested nature of our data, we used multi-level modeling for dyadic data with the software Mplus 7 (Muthén and Muthén, 1987/2017). The dataset consisted of 51 women and 51 men from 55 couples who provided 2826 reports on their daily experiences (with 9.6%, or 270 missing datapoints; missing reports were taken into account by using ML estimation). Sufficient information from both partners to estimate random variation of effects, and their covariance between partners, was available for 47 couples, but estimations for the main results reported below are based on data from 102 individuals from 55 couples. A hierarchical linear model for distinguishable dyadic diary data was estimated (repeated assessments of two partners nested within couples). In this model, repeated measures of the two individuals were represented as lower level variables, while the upper level represented between-couple variability across male partners and across female partners (Bolger and Laurenceau, 2013). Communication explicitness was coded 0 when no stressful events were experienced, such that the comparison level of explicit or implicit stress communication effects were days with average explicitness or no communication.

To examine whether explicit stress communication predicted partners’ responsive dyadic coping, which in turn predicted relationship satisfaction, we tested a within-subject mediation model, following a procedure proposed by Bolger and Laurenceau’s (2013). We controlled for the day of the diary period. The day variable was centered at day 7. We also included between-subject means of explicit stress communication. To make sure that our results referred to the effect of explicit stress communication rather than to the effects of the mere disclosure of the stressful event, we also controlled for the fact that the stressful event was communicated to the partner irrespective of how explicit this communication was. The inclusion of the disclosure variable in the model did not change the results. Disclosure, moreover, did not show any significant effect in the model. Disclosure, therefore, was not entered in the final model.

A *post hoc* power estimation for the parameters obtained in the current analyses, using a Monte Carlo procedure (see Bolger et al., 2012), suggested good statistical power for effects of explicit stress communication on responsiveness in dyadic coping behaviors (1-β > 0.84), and for effects of responsiveness in dyadic coping behaviors on relationship satisfaction (1-β > 0.97), and for women’s (1-β > 0.92), but not for men’s effects of explicit stress communication on relationships satisfaction (1-β = 0.17).

## Results

### Descriptives

Before testing the hypotheses, we examined the means and the standard deviations of all variables (Table 1). Paired sample *t*-test showed a significant effect of gender for explicit stress communication. In particular, women reported significantly higher explicit stress communication than men, *t*(46) = 2.49; *p* = 0.01 (women: *M* = 4.06, *SD* = 0.69, men: 3.56, *SD* = 0.94). Moreover, paired sample *t*-test showed that the effect of gender was not significant for perceived responsiveness in partners’ dyadic coping reactions, *t*(46) = 0.79; *p* = 0.43 (women: *M* = 3.39, *SD* = 1.06, men: *M* = 3.27, *SD* = 1.14). In addition, paired sample *t*-test showed that the effect of gender was not significant for relationship satisfaction, *t*(56) = -0.63; *p* = 0.52 (women: *M* = 4.55, *SD* = 0.71, men: *M* = 4.61, *SD* = 0.91).

**Table 1:** Values of explicit stress communication, responsiveness, and relationship satisfaction.

	Mean		*SD*		Range	
	Woman	Man	Woman	Man	Woman	Man
Explicit stress	4.06	3.56	0.69	0.94	2.5 – 5	1 – 5
communication						
Responsiveness	3.39	3.27	1.06	1.14	1 – 5	1 – 5
Relationship	4.55	4.61	0.71	0.91	3.20 – 6.27	2.21 – 6.81
satisfaction						


Finally, we examined the correlations between explicit stress communication, responsiveness, and relationship satisfaction separately for women and men. As shown in Table 2, for both partners all variables were positively correlated with each other.

**Table 2:** Pearson’s correlations between explicit stress communication, responsiveness, and relationship satisfaction.

	Explicit stress communication	Responsiveness	Relationship satisfaction
Explicit stress	1	0.34	0.30
communication		*p* = 0.01	*p* = 0.03
Responsiveness	0.51	1	0.64
	*p* < 0.001		*p* < 0.001
Relationship	0.37	0.76	1
satisfaction	*p* = 0.006	*p* < 0.001	


*Women’s correlations are above the diagonal and men’s correlations are below the diagonal.*

### Associations Between Explicit Stress Communication, Responsiveness in Dyadic Coping, and Relationship Satisfaction

**FIGURE 1:**
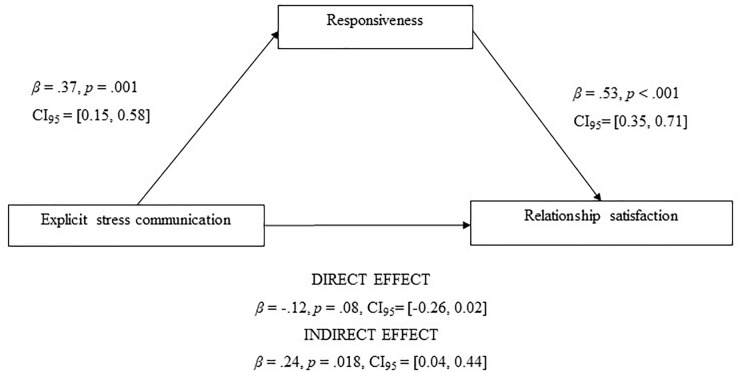
Parameters of explicit stress communication and responsiveness for relationship satisfaction.

In a series of preliminary analyses, we conducted model comparisons to examine whether the focal coefficients in the current model differed significantly between men and women. None of the tests suggested a significant gender effect [χ2 (1) < 2.66; *p* > 0.10], and we therefore set equality constraints, estimating a single set of coefficients for men and women. The results did not support the idea that more explicit communication of the stressful event was associated with relationship satisfaction, above and beyond perceptions of responsiveness (β = -0.12, *SE* = 0.07, *p* = 0.08, CI_95_ = [-0.26, 0.02]) (H1)^2^. The results showed that more explicit stress communication was positively associated with the perception of the partner’s responsiveness in dyadic coping in both women and men. In particular, on days partners communicated their stressful event more explicitly they also perceived more responsive dyadic coping reactions from their partner, as compared to days when they communicated less explicitly or when no stress was reported (β = 0.37, *SE* = 0.11, *p* = 0.001, CI_95_ = [0.15, 0.58]). Overall, these results suggested that, for women and men, communicating a stressful event in an explicit way to the partner had a positive effect on the perception of partner’s responsiveness in their dyadic coping behaviors. Findings also suggested that, in turn, responsiveness in dyadic coping was positively associated with partners’ relationship satisfaction (β = 0.53, *SE* = 0.09, *p* < 0.001, CI_95_ = [0.35, 0.71]). Finally, a test of indirect effects of explicit stress communication via perceptions of responsive dyadic coping reactions on relationship satisfaction suggested a significant mediation (H2; Figure 1; mediational path: β = 0.24, *SE* = 0.10, *p* = 0.018, CI_95_ = [0.04, 0.44])^3^.

## Discussion

The goal of the current work was to increase our understanding of explicit stress communication in the context of dyadic coping. In particular, we tested whether explicit communication of stressful events predicted partners’ relationship satisfaction, and whether perceptions of responsiveness in dyadic coping established an indirect link between explicitness in communication and relationship satisfaction. Overall, the findings underscore the role of explicit stress communication as a facilitator of perceived responsiveness in dyadic coping with daily stressors.

Disconfirming our first hypothesis, however, the findings did not reveal explicit stress communication as a predictor of relationship satisfaction. This finding is not in line with communication studies that suggest that self-disclosure is positively associated with partners’ relationship satisfaction (e.g., Sprecher and Hendrick, 2004). This points to the possibility that, unlike in a larger relational context, explicit communication of a specific experience of stressful events on a particular day is not sufficient to bring about short-term improvements of partners’ relationship satisfaction. This seems a plausible possibility, particularly since explicit communication was captured in or briefly after the stressful experience. The stressful experience itself may bear negative consequences for immediate relational well-being on its own right and therefore foreshadow the beneficial effects of explicit stress communication. Event sampling studies or longer-term momentary studies that can gather information on larger numbers of stressful events per person would allow for reliable comparisons only among different stressful episodes, which could help clarify this point. It is also possible, however, that explicit communication draws particular attention to a stressful experience, which would not only benefit the dyadic coping process, but could also lead to a more intense stress-related interaction, and therefore impede immediate improvements of satisfaction, while still facilitating improvements on a longer term. In the dyadic coping model, in fact, communication is considered as a necessary, yet not sufficient condition for the process to succeed (Bodenmann, 2005). Thus, the stress-focus of the dyadic coping situation, and the current study, may represent a case that differs from other, more general communication contexts. A study on explicit communication of *positive* events predicted both women and men’s relationship satisfaction (Pagani et al., 2015). Communicating about stressful circumstances involves more challenges and may therefore be riskier than communicating about positive ones. Disclosing one’s negative experiences may run the risk to threaten the stressed partner’s sense of efficacy and competence and therefore to elicit a negative emotional state, which could impede momentary improvements in relationship satisfaction. In negative circumstances, partners’ communication may not be beneficial *per se*, but only when eliciting a responsive reaction by the partner, as we further discussed below.

Although more explicit stress communication did not predict higher relationship satisfaction, our findings show that both women’s and men’s explicit stress communication facilitated perceptions of responsiveness of the partner’s dyadic coping behaviors. Notably, our focus was on within-individual effects. Partners who are prone to be explicit and open in communication could also be prone to perceive the other as more validating and caring, also irrespective of his/her *actual* behaviors. Our findings, nonetheless, could also reflect the possibility that explicit stress communication may facilitate the other’s *actual* responsiveness, by signaling opportunities of support and dyadic coping to the partner and by helping him/her to avoid misunderstandings, facilitate more benevolent, external appraisals of the stressed partner’s behaviors, and feel less attacked or blamed. Both of these possibilities would likely favor more responsive reactions from a helping partner during the dyadic coping process.

Finally, perceived responsiveness during dyadic coping significantly mediated the link between explicit stress communication and relationship satisfaction, confirming Hypothesis 2. Perceptions of responsiveness in dyadic coping behaviors may help each partner to experience the other as aware of and supportive to his/her needs and goals, and as willing to stand by his/her side with benevolent acceptance also in moments of difficulty and frailty (Rusbult et al., 2005; Gable and Reis, 2006; Reis, 2007; Reis and Gable, 2015). Such a sense of attention, support, and acceptance may well promote partners’ relationship satisfaction. Indeed, one partner’s perceptions about the other’s dyadic coping responses were found to mediate the association between actual dyadic coping behaviors and relationship satisfaction (Donato et al., 2015). If replicated, our findings have implications for both research on dyadic coping and for preventive interventions for couples.

As for research on dyadic coping, the present study highlights how studying the communication phase of the dyadic coping process is particularly warranted. Stress communication, in fact, has revealed as an important “situational” antecedent of dyadic coping responses as they are enacted in the context of each dyadic coping interaction. While most studies focused on individual, dispositional antecedents of partners dyadic coping responses (see for a review, Donato and Pagani, 2018), the role of more proximal and situational factors facilitating or inhibiting effective dyadic coping reactions is still under-investigated. A future line of inquiry in dyadic coping research could examine each specific component of the dyadic coping process (see also Leuchtmann and Bodenmann, 2018). At this regard, in fact, only a recent study approached a micro-analytic investigation of dyadic coping conversations (Kuhn et al., 2017).

As for preventive intervention aiming at promoting partners’ relationship satisfaction, our findings highlight two relevant aspects to be targeted by such interventions: Explicitness in communication of stressful events and partners’ responsive dyadic coping behaviors. By increasing partners’ explicitness in communication as well as fostering positive responsive reactions to the others’ communication, preventive programs aimed at training partners’ dyadic coping skills (CCET, Bodenmann and Shantinath, 2004) were found to enhance partners’ relationship satisfaction (e.g., Bodenmann et al., 2014). In particular, our findings point to the fact that the partner’s ability to make the other feel understood, validated, and cared for in stressful circumstances is of considerable importance for partners’ satisfaction. Our findings also show that the stressed partner’s ability to communicate clearly and explicitly can facilitate these perceptions and therefore the success of the dyadic coping process.

The present findings should be considered in light of important limitations of the study. The correlational nature of the effects allows for no strong causal interpretation. Moreover, the data were collected from a convenience sample of mostly non-distressed and relatively satisfied couples, and, therefore, further research with more representative samples is needed to confirm these findings for the broader population. For example, the same relation should be analyzed in couples seeking clinical help, and especially for those partners that show insecure attachment styles, which is related to ineffective support seeking and unresponsive caregiving (Collins and Feeney, 2000). In addition, our study focused on daily stressful events. We did not have information on whether such events represented minor or major stressors. Future research should test whether the associations examined in the present study would be different in couples dealing with minor stressful circumstances and in couples experiencing more severe, disruptive stressful conditions (see Randall and Bodenmann, 2009).

Although the present study tested important assumptions of the STM model, future research could profitably focus on also how explicit stress communication may be associated to specific dyadic coping behaviors by the partner. In the present study responsiveness was measured with regard to the partner’s responses to the stressed individual, while we did not measure the partner’s self-perceived responsiveness. Adding this variable in future studies could allow to test both actor and partner effects in the association examined in the present study. Moreover, cultures differ greatly with respect to communication norms and styles, in particular with regard to the emphasis on explicit communication and emotion expression (Hall, 1976; Gudykunst et al., 1996). It is therefore possible that the role of communication changes as a function of different cultural contexts. Dyadic coping, although found to be significantly associated to relationship quality across different cultural contexts, also differed in the strength of its effects across different countries (Hilpert et al., 2016). The specific cultural characteristics of the present sample may, in fact, affect the results we found. In particular, Italy presents contrasting cultural features: relatively high individualism (e.g., the value of independence and individual goals) paired with some aspects of collectivism (e.g., centrality of family of origin, widespread religious affiliation, etc.); a relatively private view of the couple relationship paired with a strong connection with familial and social ties; a traditional gender-role orientation co-existing with egalitarian expectations between partners (cfr. Donato, 2016). More specifically related to communication, Italy is a culture characterized -at least relatively to Eastern countries- by low-context communication (i.e., relying more on the explicit verbal code than on contextual cues). Thus, in Italy explicit stress communication may be more acceptable and expected than in high-context cultures, in which -instead- explicitly expressing the individual’s needs may be avoided for the sake of relationship harmony. As a consequence, in such cultures support behaviors may be offered irrespective of explicit support seeking (cfr. Falconier et al., 2016). It is possible therefore that the association between explicit stress communication and responsiveness will be weaker in high-context cultures than in low-context ones. Future research should explore this possibility. Finally, future studies may test other possible mediators of the association between explicit stress communication and relationship satisfaction, such as for example the non-stressed partner’s accuracy in understanding the stressed partner’s communication. Despite the limitations of the present study, these findings confirm an important role of communication in the process of dyadic coping. The importance of explicit communication in facilitating partners’ effective responses to the other’s stressful events confirms the active role of both partners in dyadic coping transactions. These findings call for a more attentive examination of the communication component of the dyadic coping process in both research and intervention.

## Author Contributions

AP and SD contributed equally to the research, development of the theoretical framework, performance of the statistical analyses, analysis of the results, and writing of the manuscript. MP contributed to the development of the theoretical framework and writing of the manuscript. AB and RI supervised the writing of the manuscript. DS contributed to the performance of the statistical analyses, analysis of the results, and writing of the manuscript.

## Conflict of Interest Statement

The authors declare that the research was conducted in the absence of any commercial or financial relationships that could be construed as a potential conflict of interest.

## Footnotes

1 It is important to note that not only negative events, but also positive though challenging events, can trigger coping responses (e.g., Langston, 1994; Gable and Reis, 2010).

2 Although the model popularized by Baron and Kenny (1986) proposed that establishing the relationship between X and Y is the first step in testing for a mediational effect, it is increasingly argued that step 1 (the association between X and Y) should not be an essential condition for establishing mediation (see Kenny et al., 1998; Shrout and Bolger, 2002; Zhao et al., 2010). A number of plausible reasons exist, for which the X–Y effect may not emerge as a statistically significant coefficient, even when mediation does exist: Expected and plausible multicollinearity between variables involved in a mediation; expected and plausible lower power to detect the X–Y effect than the mediated effect; etc. (see: Zhao et al., 2010; Kenny and Judd, 2014).

3 The confidence intervals were constructed based on the standard errors of the model estimates, which are based on full maximum likelihood estimation. Bias corrected confidence intervals based on a bootstrapping procedure, commonly recommended for testing mediation, are not reported because the bootstrap procedure is not available for two-level random analyses in Mplus.
